# Adaptive Intelligence: Intelligence Is Not a Personal Trait but Rather a Person × Task × Situation Interaction

**DOI:** 10.3390/jintelligence9040058

**Published:** 2021-11-26

**Authors:** Robert J. Sternberg

**Affiliations:** Department of Psychology, College of Human Ecology, Cornell University, Ithaca, NY 14853, USA; rjs487@cornell.edu

**Keywords:** intelligence, person, task, situation, interaction

## Abstract

This article explores the advantages of viewing intelligence not as a fixed trait residing within an individual, but rather as a person × task × situation interaction. The emphasis in the article is on the role of persons solving tasks embedded in situations involving learning, intellectual abilities, and competencies. The article opens with a consideration of the role of situations in intelligent behavior. The article then discusses how intelligence is more similar to creativity and wisdom, in terms of the role of situations, than many psychologists have realized. Then the article reviews the role of situations in identity-based and irrational thinking and in conspiratorial thinking and cults. Next the article discusses the demonstrated importance of situations in assessment, but also notes the difficulties in sampling situations. Finally, the article draws conclusions, in particular, that, given our lack of situation-based tests, we need to be more modest in our interpretations results from conventional tests of intelligence.

## 1. Intelligence Is Not a Personal Trait but Rather a Person × Task × Situation Interaction

Sometimes, we get so used to seeing something in a particular way that our way of seeing it becomes a presupposition—something we simply do not question. Intelligence as a personal trait seems to be that way. There are different ways of conceiving of intelligence—as a psychometric trait such as *g* or general intelligence, as an information-processing construct such as working memory, as a biological construct residing in the brain, or perhaps as a developmental program that unfolds over time (Sternberg 1990, 2020a). Even expanded theories of intelligence, such as the theory of multiple intelligences (Gardner 2011), view intelligence as a set of abilities inhering within a person. Some metaphors of mind, such as a contextual one, allow a bit more relativism, viewing what intelligence is or is conceived to be as differing from one culture to another. However, in many such accounts, intelligence is still a property of the person (Sternberg 2020b).

Metaphors of mind, such as those described above, are not inherently disconfirmable. They are perspectives on, rather than theories of, intelligence. At some level, they cannot be wrong. Certainly, intelligence does involve, at least in part, a set of abilities, processing of information, activity within the brain, and the unfolding of a developmental program. However, I suggest these perspectives of intelligence as inhering within the person may miss what may be the most important aspect of intelligence—intelligence as a person × task × situation interaction (Sternberg 2007). When people learn about the world, it is always in the context of the people performing tasks interacting with situations. This view is similar to the way in which Daryl Bem and Walter Mischel ultimately came to view personality (Bem and Funder 1978; Mischel and Shoda 1995). 

The view of this article is that intelligence is neither “in the person”, on the one hand, nor a property of everyday behavior on a given task or in a given situation. Rather, intelligence exists in the interaction between the person, task, and situation. Conventional intelligence tests sample behavior with a relatively small range of tasks—generally, measures of verbal, quantitative, and symbolic knowledge and reasoning—and within a narrow band of situations—generally, those in which individuals appear in a room for a relatively high-stakes test and are given one or more time limits to finish a set of fairly school-like tasks. However, given a broader or different range of tasks, or tested in a different set of situations, people’s intelligence might be quite different, and might be measured differently. 

For example, if people were told that the test was not only high-stakes but also that much of their future depended on their score on a given test (the actual situation with the Chinese college-entrance exam, the gaokao)—scores might be different from what they would be if the people were told that a test was being given merely to help them assess their profile of strengths and weaknesses. Similarly, scores might be different if the room in which the testing was conducted were extremely noisy, cold, hot, stuffy, or otherwise compromised. 

Some might view “true” intelligence as manifesting itself only under quiet conditions with an airy, temperature-regulated room, and a decent desk at which to work, but intelligence in the real world occurs under many different kinds of circumstances. The idea of assessment based on an analog to Whole Trait Theory (Fleeson and Jayawickreme 2015) applied to intelligence—intelligence being based upon some probability distribution of responses under different conditions—might give a better idea of a person’s adaptive intelligence than intelligence tested only in one narrow range of tasks and situations. On this view, intelligence is in the interaction of person × task × situation, and there is no single number that will well capture how people will handle different ranges of situations. Some of the people who were careless in their behavior and, as a result, got sick or died from COVID-19, might have been quite adaptively intelligent in other settings. Others who were adaptively intelligent in staving off COVID-19 may have handled poorly other kinds of situations, such as resolving interpersonal conflicts or getting their job-related tasks done in a satisfactory way.

Of course, we all recognize that people who society labels, one way or another, as “intelligent” often act stupidly or foolishly (Aczel 2019; Alvesson and Spicer 2017; Cipolla and Taleb 2021; Golob 2021; Sternberg 2002, 2018). In such cases, we view the stupid or foolish behavior as somehow an exception to the rule for the person. That is, the person is viewed as intelligent but susceptible to lapses, perhaps severe ones, in the way they act. After all, we all act stupidly on occasion, often in response to some temptation or another, or because our opinions or ideologies get the better of us. However, maybe that way of conceiving of intelligence is wrong.

I discuss the reasons for conceiving of intelligence not as a fixed trait residing within an individual, but rather as a person × task × situation interaction. I try in particular to home in on the role of the person performing tasks as relevant to situations. First, I consider the role of situations in intelligent behavior. Second, I discuss how intelligence is more like creativity and wisdom, in terms of the role of situations, than has been readily apparent. Third, I review the role of situations that call upon identity-based reasoning and irrational thinking. Fourth, I reflect on how conspiratorial thinking and thinking in cults is situationally dependent and can bypass abstract notions of intelligence. Fifth, I try to show the importance of tasks performed in various types of situations in intellectual assessment. I also, however, review difficulties in sampling situations. Finally, I conclude that tests based on tasks that do not sample situations can give us only limited information about human intelligence.

## 2. The Role of the Situation

Another way of conceiving of intelligence is as an interaction between a person with a situation, as well as with a task. On this view, intelligence is not a fixed set of internal abilities, but rather a match between abilities and a set of related situations on a particular range of tasks. For example, some people perform well under stress, but others do not; what constitutes an optimum level of stress on a given task differs from one person to another. The stress might be caused by pressure to be speedy or to be accurate or by some other stressor, but in each case, working at an optimal level of stress for an individual can enhance performance, whereas working at too high a level of stress—or too low a level—may hinder it. This is the so-called Yerkes–Dodson Law.

All views depend upon presuppositions, and this one is no different. The presupposition underlying this view is that intelligence is, at its heart, adaptive. That is, what is fundamental to intelligence is an adaptation to the range of environments with which one will need to cope (Binet and Simon 1905; Gottfredson 1994; “Intelligence and its measurement” 1921; Sternberg 2019, 2020d, 2021a; Sternberg and Detterman 1986; Wechsler 1975). 

Adaptation is a broad concept. It includes the ability to learn from the environment, to change one’s behavior flexibly in response to different environmental contingencies, and to reason well, make good decisions, and effectively solve problems within an environment. In Sternberg’s (2021a) conception, it also includes the ability to modify the environment and to select new environments in response to various kinds of circumstances. Thus, this view is broadly consistent with many different specific definitions of intelligence that have been proposed. In every case, one has to learn to deal with a wide variety of tasks as presented in an often difficult-to-predict range of situations.

This view is substantially different from the operationist view of intelligence as whatever it is that intelligence tests measure (Boring 1923; Deary 2021). Problems with this viewpoint have been extensively discussed in the literature (Sternberg 2020d). There are three major problems. The first is obvious: circularity: Tests are designed to measure intelligence, which then is defined as whatever it is that the tests designed to measure it test. The second is equally serious: It is intensely conservative and potentially impedes scientific progress. Once one has a set of intelligence tests measuring more or less the same thing, new tests will be judged as succeeding or failing by the extent to which they measure the same thing that the existing tests measure. This procedure makes it very difficult to establish a new generation of tests that measures anything other than what the old tests measure. The third difficulty is equally problematic: It tends to reduce intelligence to what is easy to measure. The options for measurement of intelligence in 1905, when Binet and Simon first published their work in France, were limited. They chose school-like tasks, which are easy to replicate with small variations. Intelligence thus was established as being based upon the kinds of learning and thinking done in turn-of-the-twentieth-century Western schools, and that is the notion that society is, for the most part, stuck on today.

The idea that intelligence in context is different from intelligence in the abstract is hardly old, but rather has been studied in some detail (e.g., Berry 1974, 1984, forthcoming; Cole 1996; Greenfield 1997; Luria 1976; Sternberg 1984; Sternberg and Preiss forthcoming; Sternberg et al. 1997). The way in which intelligence has been conceived in these works generally has been that IQ tests measure part of intelligence, but in a decontextualized way; however, intelligence is used in daily life in a contextualized way. Therefore, the poverty of contextualization of IQ tests leads to an idealized depiction of a person’s learning, reasoning, and problem-solving skills. This is what I have, myself, believed in the past.

I suggest here that this conception is wrong. The demonstration that this is wrong should have been clear from what we know based on the Flynn effect (Dickens and Flynn 2001; Flynn 2007, 2012, 2016). First, IQs rose roughly 30 points during the twentieth century in at least 14 countries (indeed, every country that was studied). Second, given that a century is too little time for genetic mutations to have taken hold, the effect must be environmental. Third, although there are multiple plausible explanations, it would seem that IQs rose to match the greater challenges that the environment (e.g., increased technology, increased education) posed as the twentieth century wore on (Dickens and Flynn 2001; Flynn 2012, 2016). That is, people’s cognitive abilities meld themselves, to some extent, to fit changing environments. An analysis of such an effect was demonstrated directly in the famous Maguire et al. (2000, 2006) studies of London cab and bus drivers. Increased use of spatial-navigation skills resulted in the growth of gray matter in the hippocampus. Taxi drivers’ learning of the complex street map of London directly affected the structure of their brains. Fourth, and most importantly, the gains were largely in fluid abilities rather than crystallized abilities. In other words, reasoning with geometric figures, which once was thought to provide a decontextualized, culture-fair measure of intelligence (Cattell and Cattell 1973; Raven et al. 1992), were anything but culture-fair or, of course, culture-free. Rather, the tests were enculturated, just in an unexpected way. Western education utilizes abstract geometric thinking a lot. Other forms of education often do not.

What early users of the nonverbal tests—and some current users as well—did not recognize is that abstract geometric symbols are not context-free or even context-reduced. They are what students learn about in many forms of Westernized preschools and K–12 schools. In the early years, such symbols are used in gamebooks and learn-to-think books for preschoolers. Later, such symbols form the basis for teaching plane and sometimes solid geometry. In other cultures, schooling is done with more concrete objects, such as those that one needs to learn to hunt, fish, gather, or, in general, to prepare for a different kind of life than that people will encounter in the post-Industrialized, technologically oriented West. 

If not even the most seemingly “culture-fair” tests are really culture-fair, what tests are culture-fair? The answer is simple: no tests (Sternberg 2020b). All tests measure adaptive skills as they are viewed from the standpoint of one or another of the available cultural contexts. Culture is a bit like an accent when one speaks and listens. One does not hear one’s own accent. However, those who grow up speaking another way do. 

For many years as a young child, I thought only Americans from my part of the United States lack an accent. Foreign speakers have their foreign accents, and then Southerners have their Southern accents, Midwesterners their Midwestern accents, and so on. We do not hear accent in ourselves, any more than we acknowledge our own cultural presuppositions. It takes people from other cultures to recognize them. For example, my wife, from a foreign country, was shocked to learn, after living for some years in the United States, that someone’s saying “Let’s have lunch sometime” often was a meaningless gesture. As a result, she often felt offended that the person never followed up. Many people born in the United States would recognize this often-empty gesture for what it is.

On this view, intelligence tests are not some kind of purified, context-free, or even context-reduced measure of intelligence. Rather, they are their own complex tasks embedded within a complex situational context that is largely hidden from those who have had post-industrialized Western schooling because the abstract, geometric context has become to us much like air or water. When we have it, we do not notice it is there.

Why, then, have IQ scores increased over the years? They have increased because societies increasingly have made the context of Western schooling important for adaptation to the environment. Indeed, today, more and more people go to college. Jobs that used to go to high school graduates now often only go to college graduates. By upping the ante with regard to the importance of Western schooling, we have more deeply embedded it into our society, from testing to schooling to job selection to job performance. Tasks appear to be context-free not because they truly are context-free, but rather because we take for granted their context so that we do not even notice the context. We take it as a “given.” The situation with contexts is much like that with accents—we do not notice accents that we hear continually. They are what we take to be normal. Other accents are measured in terms of their departure from our own accent and the accents of our peers, much as what we regard as context-impoverished is simply a context that we take to be the base context, much like the base accent. However, the base accent is as much of an accent as any other, just as the base context is as much of a context as any other. That is much the way cross-cultural intelligence has operated, and even comparative analyses of intelligence across species: Organisms are viewed as intelligent to the extent they act in enculturated ways the testers happen to value in their own cultural contexts, contexts of which they are hardly aware. The more one benefits from one’s cultural context, the less one is likely to notice it, and people entrusted to create tests for others are generally people with good jobs who have benefited from their cultural context.

Intelligence tests correlate with everyday behaviors not because supposedly acontextual measures (which actually do not exist) can well predict everyday behavior, but rather because there is transfer between complex contexts. All measures are complexly contextually embedded, whether or not it is apparent. In the past, some researchers have emphasized the differences between academic contexts and many real-world contexts, pointing out, for example, that single problems presented in academic contexts (e.g., IQ tests) tend to (a) be abstract, (b) have a single correct answer, (c) be strictly timed, (d) be emotionally uninvolving, (e) be less motivating, (f) have a clear problem definition, (g) have a clear path to solution, and (h) conform to Western cultural expectations (Sternberg 2020c). Real-world problems often have, pretty much, the opposite characteristics. Why should there be any transfer at all, as there obviously is because general intelligence correlates with so many real-world outcomes?

There is one fundamental continuity between performance in many situational contexts requiring analytical thinking—namely, that one needs to analyze the deep structure beneath the surface structure of the problem to figure out how to define the problem, how to solve the problem, and how to evaluate whether the solution one reaches makes sense. All require executive processing, or what Sternberg (2019) called metacomponential analysis. All problems in life have such an underlying structure, no matter what their context. The advantage of IQ test problems is that they are relatively easy to construct, administer, and score reliably, so that, in terms of predicting behavior in other situations, they usually will have some predictive value if there is an analytical component in the real-world problems. Moreover, most real-world problems have some kind of analytical component that requires executive functioning.

The person, tasks, and situations are typically viewed as three distinct aspects relating to intelligence, but they can be viewed as actually somewhat integrated. For example, Whole Trait Theory (Fleeson and Jayawickreme 2015), mentioned earlier, proposes that traits can be viewed as representing density distributions of states. That is, the personal trait is, loosely, a distribution of the various states one enters and how relatively frequently one enters them. For example, someone assigned the trait of “intelligent” is someone who acts more intelligently, more frequently under a variety of different situations viewed as relevant to testers’ underlying theories of intelligence. 

Others beyond Fleeson and Jayawickreme also have pointed out that intelligence has both trait-like and state-like aspects (Sternberg 2014). Moreover, in the theory of flow (Csikszentmihalyi 2008), people in a flow state often feel that their personhood merges with a task and situation, at least subjectively. In sum, person, task, and situation can seem at times to undergo at least partial mergers.

## 3. Intelligence Is More Like Creativity and Wisdom Than Theories Have Specified

There is a trio of higher-order abilities that have been studied, largely separately and as though they are distinct. The trio is intelligence, creativity, and wisdom. Although there have been attempts theoretically to integrate them (Sternberg 2003; Sternberg et al. 2021), the three constructs, for the most part, have occupied the attention of different scholars, different journals, different kinds of research projects, and different professional societies. However, this may be a mistake, because the three constructs have more in common perhaps than has been believed.

Theorists of creativity and of wisdom long have recognized that both constructs, in practice, have a strong situational component. Indeed, it is practically impossible to operationalize them in a situationally context-free way. Intelligence has been seen, at least by many psychometric, information-processing, and neuropsychological theorists, as different, and as largely independent of context. For the most part, the same intelligence tests are administered, regardless of the contexts in which people live, except, sometimes, for translations or minor adaptations, such as vocabulary, national idioms, or monetary currencies.

Creativity is typically defined as the production of ideas or products that are novel and useful (in a given context) (see definitions in Kaufman and Sternberg 2021). Clearly, this definition is contextual, and much modern theorizing about creativity is based on the notion that creativity is sociocultural in origin and conception (Glaveanu et al. 2019). An idea or product can be novel only with respect to a particular set of tasks and a range of situational contexts. Similarly, what is useful depends on the task and situational context. So, according to the standard definition, what and who is creative depends on context. That is, a person considered “creative” in one context might be viewed as pedestrian in another. For example, much of Stalinist-period Soviet art and architecture today is viewed as garish and meretricious but was not so viewed in its time, at least by those who chose to support Stalin. 

Wisdom has more varied definitions, but a common feature of many of these definitions is the seeking of a common good by balancing, over the long- as well as the short-term, various interests that will be affected by a decision (see Sternberg and Glück 2019, 2022, forthcoming). What serves a common good necessarily will vary as a function of its context. For example, almost no one thinks wars are good things. However, as a leader, one must decide whether entering a war, even though it will result in casualties, brings one closer to a common good. There is no one “right” decision. It depends on the situation. Presumably, President Franklin Roosevelt made the wise decision in entering World War II in an attempt to save the world from one of the most evil governments in the history of the world. However, President Dwight Eisenhower most likely made a mistake in initiating a war in Vietnam. It lasted many years, cost thousands of American lives, and ended up with virtually no positive results. Many other interventions, such as in Afghanistan, have proven to be no more successful, and maybe even less so, if that is possible. What is interesting, with regard to wisdom, is not that countries engage in these interventions, but rather that they do not learn from their past pattern of failures. Rather, countries, including my own, move from one failure to the next, each time hoping that things will be different without enacting or even proposing any way to make it different. 

Wisdom often is seen as a trait, and there are a number of measures of it (see Kunzmann 2019; Webster 2019), and yet there is substantial evidence that wisdom has state-like characteristics (Grossmann et al. 2019). People often are wise in one situation but not in another. The same person who is wise at work may be a fool at home. The tasks at home and at work are different, as are the situations. So, wisdom, like intelligence, seems to operate as an interaction of person, task, and situation.

Creativity and wisdom are necessarily contextually defined, in that novelty, usefulness, and the attainment of a common good, although definitionally fixed, are variable when put into practice. The nature of the common good, like novelty and usefulness, depends on context. However, how about intelligent behavior? On intelligence tests, answers are keyed as correct or incorrect, seemingly acontextually. For example, with regard to verbal/crystallized abilities, what is an antonym for “brilliant”, namely, “dull”, does not change, at least in how it is scored, as a function of context. With regard to quantitative/fluid abilities, the next number in the series “3, 5, 7, …”, namely, 9, is scored in the same way from one context to another.

One interpretation of these facts is that intelligence is somehow different from creativity and wisdom. Another interpretation is that perhaps the psychometric approach is making a category error in conceiving of intelligence as largely acontextual when, in fact, it is situationally contextual, such as are creativity and wisdom. Consider again the two problems given above.

In everyday life, one might argue, the challenge of knowing the meanings of “brilliant” and “dull” is not in knowing they are antonyms, but rather in knowing how to judge them. What does it mean, say, for a work of art or music or poetry or scientific research or anything else to be brilliant, and what does it mean for it to be dull? What does it mean for a person to be bright or dull? For an engineer, what does it mean for a surface to be brilliant or dull? What or who is “brilliant” in one context may be “dull” in another context, and vice versa. The IQ test asking for the antonym barely scratches the surface of understanding, looking only at its most superficial aspects. That is what an IQ test does—measure somewhat superficial thinking skills. Such skills are not trivial; they are just not enough to get one through life. 

One would hope that there would be, in a quantitative test, more of a definitive measurement than in a verbal test, but is there? Number series and the large majority of tests of fluid abilities measure inductive reasoning. By definition, inductive-reasoning problems do not have a definitive answer. Unlike in deductive reasoning problems, such as syllogisms, there is no one answer that is logically correct. If “All men are mortal and Socrates is a man, then Socrates must be mortal”. End of story. However, induction problems, the cornerstone of tests of fluid intelligence, have no such definitive answer. Number series have an infinite number of possible completions, depending upon the formula that generates them. This is easy to see with the example given. Although “9” might the “correct” response if the sequence is one of integers ascending by 2, the correct answer would be “11” if the “correct” response were successive ascending prime numbers. There are infinite other “correct” answers as well, depending on the underlying rule.

Peter Wason (1968) has made a similar point in his studies of the “2, 4, 6…” problem. Wason presented participants in his research with these three numbers and asked the participants to formulate the rule underlying the numbers. Almost all of the participants guessed that the rule is, essentially, ascending even numbers starting with two. However, that was the wrong rule. So, Wason asked them to keep trying to figure out the rule. The participants came up with many rules but rarely came up with the correct rule. That was because they did not try to disconfirm their own false assumption, namely, that there must be a fairly specific rule rather than a general one. The rule Wason had in mind was increasing integers—any increasing integers at all. However, most participants kept trying out hypotheses that were more specific and even that they previously had identified as incorrect. Like the US presidents, but with far lower stakes, they failed to learn from experience. 

The college-student participants were bright, so why did they keep failing? Why do US presidents continue in failed foreign ventures and disgraceful exits?

Sternberg (2021b) has proposed a relevant theory of information representation and processing in intelligent thinking. The basic idea is that there are two principal processes—seeking of external correspondence and of internal coherence. Seeking of external correspondence refers to seeking knowledge about what is taking place in the external world. In order to think and behave intelligently, we need to understand the environmental situations in which we find ourselves. What is actually going on? 

Seeking of internal coherence refers to seeking connections among the pieces of knowledge that one has—seeing how they fit together and provide larger meaning. Simply receiving information is not really all that useful. What matters is what the information means in the context of all the information one has and that one can acquire. One needs selectively to compare the information one has to past information available from all possible sources and also selectively to combine the information one has so that it makes some kind of sense (Sternberg and Lubart 1994). 

At a superficial level, tests of crystallized ability measure the success of a person in having sought external correspondence; tests of fluid ability measure the success of a person at finding internal coherence—at successfully seeking out patterns. However, as we saw above, such tests measure only superficial aspects of these processes. Such thinking is not unimportant, but it also will not solve consequential real-world problems. 

Seeking of external correspondence and internal coherence speak to the importance of intelligence in action. When either is out of balance with the other or suppressed, individuals are susceptible to irrational and even cultlike and reasoning as well as fantasies of wild conspiracies. 

## 4. The Interactional View in Action

Consider two applications of the view of intelligence as interacting with a situation as well as the range of tasks confronted in various situations.

## 5. Identity-Based and Irrational Thinking

In the extreme case, suppose someone scores at a gifted level on an IQ test, and then, knowing the predictable consequences, immediately acts in a way that predictably results in their own death, despite their wanting to live. Were they truly intelligent? If so, what, exactly, does intelligence mean—the ability to recognize antonyms and complete number series and then to act so stupidly that they cause their own unwanted death? Is not intelligence the ability to adapt to the environment?

There are any number of examples one could give to illustrate the point. Where does one start?

Ron DeSantis, Governor of Florida in 2021, when this essay is being written, was graduated from Yale University with a bachelor’s degree and from Harvard Law School. It is hard to have more impressive academic credentials than that. DeSantis has issued an executive order in Florida that forbids public schools to mandate masks in school classrooms. His state ranks #3 of 50 in COVID-19 cases (https://www.statista.com/statistics/1102807/coronavirus-covid19-cases-number-us-americans-by-state/). The US Centers for Disease Control recommends masking in schools (https://www.cdc.gov/coronavirus/2019-ncov/community/schools-childcare/k-12-guidance.html; accessed 23 November 2021). The CDC’s goal is to prevent sickness and death. DeSantis’s proclamation says that his goal is to protect individual freedom. The problem is that people do not have the freedom to do a lot of things that they might want to do if those things hurt others—to drive drunk or to shoot people whom they find to be undesirable or offensive. If a young child contracts COVID-19 and breathes on another young child, the first child may inadvertently cause illness or death in the second child simply because of being unmasked. What is hard to understand about this scenario? Countries understood it with smallpox, polio, and other diseases. Is COVID-19 different?

DeSantis, like many others in government, may be cynical and decide that his political future is more important to him than children’s health. Or he may truly believe that the CDC is wrong, although it is unclear why a politician with no medical education would know more about the transmission of illnesses than experts in the field. This is not a matter of politics, but rather a matter of public health and saving lives, which are, or at least should be, apolitical. Governor Abbott in Texas, whose state ranks #2 in COVID-19 cases (as of 23 November 2021), has issued a similar order forbidding mask mandates.

Herman Cain was graduated from Morehouse College, one of the most prestigious Historically Black Colleges and Universities (HBCUs). He majored in mathematics, one of the hardest majors in any college or university. Cain grew up in challenging circumstances, so his admission to Morehouse was all the more impressive. His mother was a cleaning woman and his father was a janitor, barber, and chauffeur at various points. Nine days after attending a political rally maskless, Cain was diagnosed with COVID-19 (https://www.reuters.com/article/us-health-coronavirus-usa-cain/herman-cain-ex-presidential-candidate-who-refused-to-wear-mask-dies-after-covid-19-diagnosis-idUSKCN24V2OD; accessed 23 November 2021). One cannot confirm that Cain contracted COVID-19 at the rally. But, in fact, he died shortly after attending it maskless. His obvious “raw intelligence” was not enough to save him from the kind of poor judgment that is leading millions of people to fail to take precautions and thus to get sick and sometimes die from an at least partially preventable disease. The individuals may be intelligent in the abstract on an IQ test, but how much does it matter if they act in a suicidal (infecting themselves) or homicidal manner (infecting other people)? 

There are other ways as well that people who are viewed as “intelligent” act that might lead one to question whether intelligence simply resides within the individual.

## 6. Conspiratorial Thinking

### 6.1. What Is Conspiratorial Thinking?

Conspiratorial thinking is an example of how intelligence in action in specific kinds of situations can go far astray from the kinds of thinking measured by intelligence tests. Because of the severe entrenchment in the field, people are viewed as “intelligent” even when their thinking unintentionally leads fairly directly to their death and the death of others, so long as their IQ is intact. Somehow, in the years since the work of Alfred Binet, the meaning of *intelligence* has been largely trivialized and, arguably, lost.

Cult leaders, whether of religious, ideological, or political cults, do their best to suppress their citizen’s intake of factual information about the world and even to control their language (Montell 2021). Instead, the leaders often provide false information to their followers that basically makes life in the confines of their cult look good and life in other settings look bad. It is impossible to think in a truly intelligent way because the knowledge base on which one is drawing, and the language used to describe it, are defective. One may think intelligently—but the thinking is flawed because it is based on an erroneous knowledge base (flawed external correspondence). The leaders not only try to suppress information but try to hide that they are suppressing information.

Cult leaders indoctrinate their followers with doctrine, rituals, and apocryphal stories present as fact in order to ensure that followers draw the connections and establish the internal coherence that the cult leaders want them to draw. Sometimes, members of the cults are put into situations of high pressure and deprivation to reduce the quality of their critical thinking and their ability to draw their own independent connections.

When the quality of both one’s external correspondence and internal coherence is destroyed, people even with high levels of general intelligence can end up thinking in irrational ways. Moreover, if they do not know of the degradation of their own thinking, they may view their own thinking as quite intelligent.

Thus, people can be intelligent, in a conventional sense, but nevertheless fall prey to irrational, cultlike thinking through the manipulation by cult leaders of the people’s (a) knowledge providing external correspondence and (b) mechanisms for providing their own internal coherence to what they know.

Conspiracy theories have five essential elements (van Prooijen 2018). The first is *patterns*—the believer sees a pattern, usually one that he or she believes eludes many other people. The second is *agency*—whatever is going on is being believed to be caused purposely by agents. The third is *coalitions*—there are multiple actors forming the conspiracy; the multiple actors are what make it a conspiracy. The fourth is *hostility*—the actors have threatening, hostile, evil, or malign intentions, or some combination of these. The fifth is *continued secrecy*—the evil-doers are operating in secret to facilitate their malign intentions and plans. 

### 6.2. Who Believes in Conspiracy Theories?

The number of conspiracy theorists and even theories is uncountable—for example, that John F. Kennedy was killed as a result of a conspiracy; that Donald J. Trump lost the election in 2020 because of a conspiracy; or, according to Trump, that a conspiracy existed to hide the fact that Barack Obama was born in Kenya. Some believed that Hillary Clinton was part of a conspiracy of satanic child-molesters. The conspiracy, informally labeled Pizzagate, gained many believers, despite its utter ridiculousness and lack of evidence, resulting even in Edgar Maddison Welch forcing his way into a restaurant with a gun in hand in search of the captive children in the basement. Actually, the restaurant had no basement (Robb 2017). 

Maybe Welch had a low IQ. We do not know his IQ. However, how about the 53% of Republicans in the United States who believe that Donald Trump is still the “true president” of the United States (Guardian Staff 2021)? The conspiracy theorists (and cynics who did not really believe the conspiracy) filed over 50 lawsuits (63, to be exact) against the election. Many of these lawsuits were heard by Trump-appointed judges. All but one were dismissed without going to trial (Reuters Staff 2021). The one that was not dismissed was dismissed by the Pennsylvania Supreme Court. Perhaps the court system is part of the conspiracy, including even Trump-appointed judges.

Conspiracy theories are not limited to the political right-wing. Many anti-vaxxers are left-wing. One left-wing conspiracist has claimed that Bernie Sanders, a former US presidential candidate, is actually an agent of Vladimir Putin (Bernstein 2017). A 2012 national poll in the US found that 37% of Democrats were convinced that President Bush’s supporters won Ohio in 2004 by committing fraud; 36% of Republicans believed that Obama’s supporters committed significant fraud during the presidential election of 2012 (Moore et al. 2014). For whatever reason, conspiracy theories, such as that the belief that human-induced climate change is a conspiracy, occur more often on the political right and especially those who are low in conscientiousness (Lawson and Kakkar 2021), despite the belief of more than 97% of climate scientists—who, unlike the conspiracy theorists, actually scientifically study climate change—that humans have induced global climate change (NASA Global Climate Change n.d.). The catastrophic fires and weather events of 2021 seem to have little effect on conspiracy theorists regarding climate change, and indeed, facts are largely irrelevant to conspiracy theorists, as targets, such as George Soros, have discovered. Soros, an international financier, has been the target of many anti-Semitic conspiracy theories, despite the lack of evidence for any of them (Cohen 2020). 

### 6.3. Why Do “Intelligent” People Believe in Conspiracy Theories?

In 2015, it was estimated that about half of Americans believe in at least one conspiracy theory (Sides 2015). However, that was then. Today, almost two-thirds of Republicans believe in at least one QAnon conspiracy theory (Swan 2021). Over 20% of Americans today believe in at least one of four COVID-19 conspiracy theories: that COVID-19 vaccines (a) contain microchips allowing people to be tracked; (b) can alter a person’s DNA; (c) contain lung tissue derived from aborted fetuses; or (d) can cause infertility (Beeri 2021). Why is there no empirical evidence of any of these claims? That is obvious. There is also a conspiracy to hide the truth!

Why do people believe conspiracy theories? According to van Prooijen (2018), people are more likely to believe conspiracy theories when they perceive there to be a societal crisis. When there is a perception of a big crisis, they look for a big cause, namely, a conspiracy. When they experience fear and uncertainty, they are likely to look for patterns, and they even imagine patterns that do not really exist. 

There are two major processes involved in the detection of conspiracies, false or real (and indeed, some conspiracies are real): pattern perception and agency detection. People start to see patterns, which may or may not exist, because they need to make sense of events that seem to them inexplicable. Then they need to find the agents who are responsible for the creation of these patterns, that is, the conspiracists.

In terms of Sternberg’s (2021b) AWOKE theory of information processing in intelligence, pattern recognition is part of seeking internal coherence and agent recognition is part of seeking external correspondence. Put another way, conspiratorial thinking calls upon both aspects of intelligent thinking. When intelligent thinking goes awry in a conspiracy theory, it is because it is erroneous in detecting a pattern that does not exist (or distorting a pattern that does exist) and/or it is erroneous in assigning agency.

The question with which intelligence theorists need to grapple with is whether theories and measurement of intelligence should focus on contexts that are designed to be as independent as possible of external distorting factors, or whether to recognize that these external distorting factors always exist, to some degree, in life, and that therefore one needs to take them into account in understanding and measuring intelligence. That is, do we want to measure intelligence as it exists under conditions that are not likely to be replicated quite anywhere except during testing, or do we want to measure intelligence under a variety of conditions that actually exist in people’s lives? An excuse might be that the tests only should measure some kind of “pure intelligence”, but the whole point of this essay is that there is no “pure intelligence” or pure intelligence test, except in the minds of some of those who devise tests and of others who have bought into the same myth.

## 7. Adaptive Intelligence

We might call the result of such a measurement *adaptive intelligence*—one’s level of intelligence as it actually exists to be deployed to adapt to the situational conditions one faces or is liable to face in one’s life (Sternberg 2019, 2021a). One may or may not be successful in such deployment, but one’s adaptive intelligence represents a person’s ability to cope with the person’s actual and likely tasks in a variety of potential environmental situations.

Adaptive intelligence changes with time and place. How well people can establish external correspondence and achieve internal coherence always will depend upon situational factors. There are no “pure” measures because even intelligence tests comprise tasks are administered to persons in particular kinds of situations—situations more familiar to those with advanced Western educations than to many others. There is no one test that measures adaptive intelligence because people differ not only in their skills, but equally importantly in their flexibility in deploying these skills *adaptively* under different situational constraints. Some of my colleagues and I are working on tests of adaptive intelligence, but with what we hope is the humility to realize that the the tasks may need to be radically modified to fit different kinds of situational circumstances. 

Indeed, others have recognized this variability in the nature of intelligence. As noted earlier, Berry (1974, 1984) was among the first to recognize that different cultures have different conceptions of what it means to be, and need different things actually to be, intelligent. Greenfield (1997) argued that “you can’t take it with you”—that the intelligence you bring from one culture often does not transfer well to another. She further argued (Greenfield 2019) that the nature of intelligence changes over time—that what societies require differs as they become more technologically oriented. So, intelligence is not a fixed, context-free quality, but rather one that varies with time and place.

## 8. Sampling Situational Contexts, Not Just Persons and Tasks

From the point of view of the present essay, current procedures for testing intelligence and related abilities address two problems without addressing a third equally important, and perhaps more important one.

**Persons.** When contemporary intelligence tests are normed and validated, the constructors of the tests need to sample a large number and a wide variety of persons to ensure that their sample is representative of the population to which the results of the sample are supposed to generalize. Without such sampling, any norms and validation evidence will be suspect. Often, initial standardizations are somewhat limited in their sampling, and some tests are never standardized on broad swaths of the population.

**Tasks.** At the same time, to ensure validity, contemporary intelligence tests sample performance across a range of cognitive tasks, for example, perhaps verbal, quantitative, and figural ones. 

**Situations.** Where contemporary tests of intelligence typically fail is in their sampling of situations. Intelligence in real life occurs under conditions that typically resemble only to a modest degree the conditions under which tests are administered (Sternberg 2020c). People need to learn not only how to deal with different kinds of tasks, but also with the different kinds of situations in which the tasks can be confronted. Learning to deal with diverse situations is part of responding intelligently. Moreover, we know that situations greatly affect task performance. 

Ceci and Bronfenbrenner (1985) found that context had a major effect on performance in prospective-memory and time-monitoring tasks. Nuñes (1994) (see also Ceci and Roazzi 1994) found that context affected street children’s ability to perform mathematical tasks. Grigorenko et al. (2004) found context effects on the ability of Alaskan children to solve problems, as did Sternberg et al. (2001) for Kenyan children. In this latter case, tests of adaptive intelligence in a rural Kenyan context correlated negatively with scores on IQ tests. Preiss et al. (2016) found that the type of school where the participants studied (university versus technical school) moderated the effect of metacognition on creativity.

It might help to give a couple of examples that have served to measure adaptive intelligence in contexts other than that of conventional standardized tests, the first from Sternberg et al. (2001) and the second from Grigorenko et al. (2004) (with correct answers asterisked):

1. “A small child in your family has homa. She has a sore throat, headache, and fever. She has been sick for 3 days. Which of the following five Yadh nyaluo (Luo herbal medicines) can treat homa?
  - Chamama. Take the leaf and fito (sniff medicine up the nose to sneeze out illness).*
  - Kaladali. Take the leaves, drink, and fito.*
  - Obuo. Take the leaves and fito.*
  - Ogaka. Take the roots, pound, and drink.
  - Ahundo. Take the leaves and fito.’’
2. “When Eddie runs to collect the ptarmigan that he’s just shot, he notices that its front pouch (balloon) is full of ptarmigan food. This is a sign that:
  - there’s a storm on the way.*
  - winter is almost over.
  - it’s hard to find food this season.
  - it hasn’t snowed in a long time.”

These items are somewhat typical of “emically”-derived items used to measure intelligence as adaptation, that is, items that arise indigenously from a culture rather than items that are based on one culture’s conception and then carried over (or imposed) on another culture (“etically”-derived items). For children in rural Kenya, knowledge of how to recognize and treat parasitic illnesses is of paramount importance. Malaria, for example, is one of the world’s great killers, having killed between 150 and 300 million people in the 20th century (Carter and Mendis 2002). 

The ability to prevent malaria might seem like a culture-specific ability, but we have seen in the 21st century that prevention of disease is a very contemporary problem of adaptive intelligence all around the world today. COVID-19 has killed over 5 million people in just a couple of years, and many of these deaths could have been prevented by adaptively intelligent strategies, such as social distancing, mask-wearing, and vaccination. Some people, of course, cannot use these prophylactic measures. However, others can and do not. Some of them end up sick and a subset of those end up dead. If adaptive intelligence is not about the preservation of one’s own life, what then is it about? Many of those who were unvaccinated and died had children who, through their parents’ probably unnecessary death, were deprived of parenting they otherwise would have had.

The point is that what is “intelligent” interacts with the kinds of tasks the environment in which one is situated presents to the person. The ability to head off diseases seemed to be largely on the way out as an important aspect of intelligent performance at the beginning of the era of antibiotics, but it never in fact quite disappeared. With the advent of COVID-19, which is one of many pandemics in history, some people who were adaptive to their environment realized the need for precautions. Others, blinded by ideology, “fake news”, entrenchment, or sheer ignorance, ignored the rapid change in the environment, in many cases at their peril. 

The problem is not limited to pandemics or to rural African environments. With the end of Communism, what was adaptively valuable in the environment of the new Russia was very different, at least initially, from what was adaptively valuable during the Communist regime (see Grigorenko et al. 1997; Grigorenko and Sternberg 2001). Academic intelligence became much less important and practical intelligence much more important, because the academically oriented jobs, in many cases, stopped paying, or started paying very little. Should a society suddenly become a dictatorship, as has recently happened in Hong Kong, very different skills are needed to survive from those skills that flourish in a free society. What would have been adaptively intelligent in one moment may land one in prison for a long time or for life in another moment, with the difference sometimes just a matter of a day. Venezuela and Nicaragua also relatively quickly went from democracies to dictatorships, with new skills needed for adaptation and even survival.

The upshot is that there are many environmental effects (Flynn and Sternberg 2020) and cultural effects (Sternberg 2020b) that alter scores on intelligence tests and that affect performance on tasks requiring intellectual investment. Contemporary intelligence tests, for however well they may sample persons, generally sample a limited range of tasks and situations not at all, as though the situations do not matter. However, we know that situations matter.

Table 1 lists some of the kinds of situations that can affect how intelligently we perform tasks. These include, in no particular order, constraints of (a) time, (b) accuracy, (c) stakes, (d) emotions, (e) identity, (f) resources, (g) mood, (h) health, and (i) pharmacological state. This list is by no means complete. For intelligence testing to be fully meaningful, it would need to sample situational constraints as comprehensively as it samples types of persons and tasks. The fact that contemporary intelligence tests do not sample situations and the full variety of tasks needed for success in those situations may be why intelligence tests have remained largely stuck in their validity: Validity coefficients are little or no higher today than they were in the past, because the tests are largely the same. (If the validity coefficients sometimes appear to be higher, it often is because of corrections for attenuation and restriction of range.)

If situational constraints affect intelligent performance, why have situational constraints not been sampled, then? I suggest there are several reasons:

1. **Ease of current measurement.** It is easy to administer conventional standard IQ tests.
2. **Difficulty of situational measurement.** It would be challenging to administer intelligence tests where testers varied situational constraints.
3. **Cost of measurement.** It would be far more expensive to conduct situational measurement.
4. **Time of measurement.** It would take longer to conduct situational measurement.
5. **Viability for participants.** It might be difficult to persuade many people to take tests in which they are challenged emotionally, ideologically, pharmacologically, or in terms of their identity.
6. **Complacent conservative theorizing—entrenchment.** Psychometricians and many of their followers have become comfortable with the existing tests.
7. **A little success is taken as a lot of success.** The success of the tests is viewed by some as sufficient, even though it is far from perfect.
8. **Finding a place to administer the situational tests.** Assessment centers might be able to handle some aspects of situational testing, but it is not clear that the standard psychologist’s laboratory, clinical settings, or educational settings would be able to do so.
9. **Profitability.** The existing tests have been enormously profitable for test publishers and for others that capitalize on the market for helping students and others to prepare for the tests.
10. **Superstition.** Some people have a belief that seems almost magical that the tests tell one what one needs to know and that they are comprehensive and even complete in their assessment of what matters for intelligence. For the most part, they have not sought to disconfirm this belief by trying to live without the tests, although COVID-19 has forced some colleges and universities to do exactly that.

Thus, intelligence might best be measured for persons as an average task performances subject to weighted situational assessments, with weights assigned by the frequency with which the situations are confronted; but the practical obstacles are indeed daunting. 

## 9. Conclusions

I have argued in this article that intelligence is an interaction between a person, tasks, and a wide variety of situations. Thus, stated fully, intelligence is a person × task × situation interaction. 

Others have shown the importance of such interactions. For example, Danner et al. (2011) showed that dynamic decision-making can predict supervisory ratings beyond the prediction provided by IQ. Rauthmann et al. (2015) have provided a taxonomy of kinds of situations and how they affect cognitive and other kinds of performance. Ziegler et al. (2018) have shown how, in task performance, openness to experience and interest interact with each other. These are only a few of the many studies that have shown that measuring intelligence or other abilities is best done in the context of person × task × situation interactions.

Although some articles have claimed that intelligence does not show much situation-specific variation (e.g., Danner et al. 2011), this may be because of severe restriction of range in the number of tasks and/or situations that have been sampled. Using a broader range of persons, tasks, and situations that go beyond the individuals and the individual educational and vocational opportunities of mostly middle- to upper-middle-class Western participants, as described earlier in the exposition of cultural work (see Sternberg 2020b), might present a rather different picture. Studies in diverse cultures with broader conceptions of emotional, social, practical, and cultural intelligence suggest there may be more to the picture than just general intelligence (Ang et al. 2020; Hedlund 2020; Kihlstrom and Cantor 2020; Rivers et al. 2020; Sternberg 2019).

Psychologists can measure intelligence as a fixed entity, as they typically do with intelligence tests and their proxies. However, such measurements are incomplete, arguably, dreadfully incomplete, because all we have to do is look at the state of the world to see how limited performance on intelligence tests is at predicting real-world success. We have plenty of high-IQ leaders who continually make the messes of the world even messier. They work well within the range of tasks presented on IQ tests but operate sub-optimally as the range of tasks and especially situations expand beyond that in the tests. 

Markus and Borsboom (2013) have pointed out that tests can be seen either as behavioral manifestations of underlying latent psychological constructs or as behavioral samples over a specified range of situations. Each interpretation has both advantages and disadvantages. For example, it is comfortable for a psychologist, given their training, to think in terms of underlying causal constructs; but it is also comfortable, in a different way, to have the assurance that one can specify just what it is, behaviorally, that a test score represents, minimizing or eliminating reference to hypothetical internal constructs.

The view presented in this article represents something of a middle ground, or perhaps a third ground. It views there being a developed set of abilities residing, at some level, within a person. In this way, the theory would have something in common with theories such as that of Carroll (1993) or McGrew (2005. That said, these abilities are not conceived of in terms of psychometric factors, but rather in terms of metacomponential (executive) processes—recognizing the existence of a problem, defining the nature of that problem, allocating resources to the solution of the problem, mentally representing the problem, formulating a strategy for solving the problem, monitoring problem solving, and evaluating problem-solving. Although these processes are relevant cross-culturally and across different epochs in history, how they are applied—in terms of the kinds of tasks to which, and situations in which they are applied—can vary greatly. As a result, the person × task × situation interaction can vary, and who is considered to be “intelligent” can vary widely across different tasks and situational contexts. Indeed, many of us have traveled abroad and found that our skill levels for coping are distinctly lower than they were in the environments to which we have become accustomed.

It is much easier to talk about the theoretical possibilities for measuring intelligence situationally than to operationalize these possibilities. Even one situational manipulation can require a major effort on the behalf of the tester and possibly of the test-constructor as well. So, tests that measure intelligence across a range of situations people confront in their lives may be beyond our grasp at the moment, unless our technology and possibly our command of measurement in virtual reality improves. There is one thing we can do right now, however, and that is to recognize the limitations of current assessments. We need to go beyond culturally sanctioned, individualistic criteria for the success of tests, such as grade-point average, years to graduate from college, the prestige of college or job, etc. (Sternberg 2021a). We need to look at the criteria that matter for keeping the world on a path toward long-term survival. Many of these criteria are collective and need yet to be worked out. Our greatest problem for now, then, may be to realize the limitations of what we do in our assessments, so that we do not draw conclusions about the potential for success that go way beyond what the data tell us.

Intelligence, experts agree, comprises skills of learning, thinking, and adaptation (Sternberg and Detterman 1986). It is a primary basis of many kinds of cognitive competence. Intelligence tests measure these skills in a superficial fashion. If we look at responses to COVID-19, especially in some of the states that are having explosions in numbers of delta-variant cases in November 2021, it is hard to see where learning, thinking, and adaptation are operating. Countless lives are being lost as a result of innumerable people not doing much of any of these to combat the delta-variant. We need better to understand how we can recruit intelligence to solve real-world problems, not just IQ-test ones. It is in a way puzzling that, in the intelligence business, many scholars are more concerned about how people solve problems on IQ tests than about how people solve problems that might save lives, including their own. The notion of intelligence as adaptation, dating back to Binet and Simon (1905), seems, at times, to have gotten lost. Perhaps it is time to realize that whatever a person’s IQ score, what will matter in their lives is the person × task × situation interaction in the nature, activation, and deployment of their intelligence.

## Figures and Tables

**Table 1:** Situational variables that can affect intelligent thought and action.

Category of Constraint	Nature of Constraint	Example of Constraint
**Time Constraints.**	The participant is told that they have limited time to complete a task or test.	One is told to do a task within a certain amount of time that seems to the individual insufficient for completing the task.
**Accuracy Constraints.**	The participant is told that their score on a task or test depends upon accuracy.	One is told that if one makes a mistake (e.g., a surgeon), someone (e.g., a patient) may die.
**Stakes Constraints.**	The stakes on a task or test are particularly high and even crucial to one’s life.	One is told that a bad decision may lead to one’s financial ruin.
**Emotional Constraints.**	The task or test generates strong emotion that interferes with, or possibly facilitates, performance.	One finds that a task of figuring out how to extract a loved one from a bad intimate relationship provokes so much consternation that one cannot think straight.
**Identity Constraints.**	The task or test somehow either threatens one’s personal identity or else possibly enhances that identity.	One is asked to perform a task that one perceives as hurting members of one’s own tribe, such as writing a persuasive essay directed against practitioners of one’s own religion or political party.
**Financial or Other Resource Constraints.**	One is unable to do the task for lack of adequate resources.	One is asked to do a task that would require payments to others, but for which one does not have the money to pay those others.
**Mood Constraints.**	One’s mood facilitates or impedes one’s ability to complete a task or test.	One is extremely depressed but is asked to finish a difficult mathematical task nevertheless.
**Health and Other Physical Constraints.**	One’s health or other physical constraints impede or possibly facilitate one’s ability to complete a task or test.	One is asked to undertake a challenging physical task, despite the fact that one is too ill to complete or even try to accomplish the task.
**Pharmacological Constraints.**	One is impeded or facilitated by pharmacological substances one has ingested.	One has drunk a lot of caffeine to stay alert during a task; alternatively, one is asked to do a complex cognitive task while under the influence of alcohol.

## Data Availability

Not applicable.
